# Assessment of solar e-cookers social acceptance in Gaza Strip

**DOI:** 10.1038/s41598-022-22326-6

**Published:** 2022-10-14

**Authors:** Hala J. El-Khozenadar, Tamer Khatib, Besan Attaee, Rifa J. El-Khozondar

**Affiliations:** 1 Department of Electrical Engineering and Smart Systems, Islamic University of Gaza, P.O.Box 108, Gaza, 860 Palestine; 2 Department of Energy Engineering and Environment, An-Najah National University, Nablus, 97300 Palestine; 3 Department of Physics, Al-Aqsa University, Gaza, 860 Palestine

**Keywords:** Psychology and behaviour, Photovoltaics

## Abstract

This study aims to assess the social acceptance of using solar energy based cooking appliances in Gaza Strip. A study sample that consists of 2400 employees from three local universities in Gaza strip is targeted in study. Meanwhile, 347 participations have participated in the study. This gives the conclusions of the study a margin of error of 5% and a confidence level of 95%. Different attributes are used to measure the social acceptance of the respondents of solar energy cooking systems including knowledge of using solar cooking appliances, financial situation, educational level, age, career and gender. According to the results, 94.55% of the participants believe that the best usage of solar energy is for lighting. Meanwhile, only 37.7% of the participants have supported the usage of solar energy for cooking. It is also concluded that there are no statistical significant differences in using solar energy for cooking associated with gender and job status. Meanwhile, it is found that there is statistical significance of using solar energy for cooking associated with education and age. This shows a clear behavioral barrier for the usage of solar energy cooking systems in Gaza Strip. According to this research it is concluded that end-users with middle income put quality of life first before the technology cost (affordable costs). This conclusion is regardless the educational level of the respondents. Moreover, it is found that Funding schemes and loans are key issues in spreading the e-cooking. Finally it is concluded that noticed support of using solar energy in Gaza according to this research is directly associated with energy poverty status in Gaza. Meanwhile, the idea of using green alternative energy is very acceptable in Gaza but there is a clear lack of awareness of technologies aspects and characteristics.

## Introduction

In low-income counties, people usually use primitive methods for cooking like dung, wood and crop waste to fulfil their energy needs instead of using traditional fuel^1^. In Gaza Strip, the energy situation is complicated due to the political situation^2,3^. People in Gaza Strip suffer from electricity shortage due to the siege imposed on Gaza Strip since almost 14 years. Thus, there is a good motivation to use renewable energy sources as a replacement for traditional energy. As a matter of fact, solar radiation can be used either directly or indirectly by electricity generation methods for cooking food as an alternative to fuel, wood, kerosene and other types of fuels^1^. Thus, solar energy has the potential to improve the socio-economic status by reducing the time and effort that are spent on cooking, and mitigating the health risks associated with current cooking practices^4^. As a fact, researchers found that solar cooking methods offer an attractive alternative to many problems that are caused by the unsustainable use of biomass^5^. Furthermore, improving people’s access to modern energy services can make a big difference in people's life quality^6^.

Studies show that user positive experience helps in developing future generation of the technology as well as may increase the usage of technology^7^. In^8^, the researchers found that the solar-powered box-cooker is the best alternative for low-income families in developing countries. Moreover, it is stated in^8^ that solar cooker has many benefits to the environment, economy and health. It is also concluded in^8^ that there are many variables that may affect the use of solar-powered cookers. These factors are economic factors (related to cost); social factors (characteristics of suppliers); cultural factors (food and daily routine); environmental factors (solar energy potential, availability of alternative fuel, place of cooking, level of infrastructure); political factors (policies to adopt solar cooker); and technical factors (ease of use and availability of cooker parts)^8^.

In^9^, the researcher conducted interviews with families in three different locations that have solar-powered cookers. The results of the interviews revealed many factors that affect the use of the solar cooker including social and cultural factors, these factors include food culture; climatic situation; economic factors and the price of the solar cooker; box cooker quality and its durability; accessibility; accessories; operation and maintenance cost and access to information. In addition, it concluded in this study that there are several things that may limit the success of solar cooking such as economic costs, social and cultural factors, information, infrastructure, technology, sustainability, and location.

In addition to that, factors that affect user's decision to use solar cookers have been presented in^10^. The authors categorized these factors through different stages which are knowledge, persuasion, decision, implementation and formation. According to this study, the awareness and understanding of solar cookers among consumers is a crucial precondition for being able to make a decision about using it. In order to adopt the system, users must be aware of the benefits of solar cooking in order to form a positive attitude about solar cooking. In addition to that, in^11^ it is reported that by using a solar cooker, many advantages in terms of energy delivery and time of cooking can be achieved. It is also reported that even low income people become users of solar-powered ovens. However, the high initial cost of cookers is still considered as a disadvantage. This is because some of the systems include solar panels and batteries which increase the cost of solar cooker^1,11,12^.

In all of these previous studies, it was concluded that users with low-income cannot invest in energy-efficient technologies due to the lack of awareness of energy costs and pay back periods. Meanwhile, it is found that accessibility and cost are the main reasons for choosing fuel for cooking and heating. It is also reported that the cooking methodology is determined by families by considering efficient use of food and fuel resources as well as food safety. These reasons can be related to environmental issues, marketing and price factors in relation to different attributes like income and job title.

Gaza Strip is located in an area that is considered one of the most exposed regions in the world to solar brightness. As a fact, Gaza is located in the sun belt with more than 300 sunny days per year and an average of 8 h of daily brightness with annual average solar irradiance ranging from 5.4 to 6 kWh/m^2^ day^13^. These facts motivate this work which attempts to assess the use of power cooking appliances in Gaza. Thus, the objective of this paper is to investigate the factors that affect the decision in adopting renewable energy practices in areas that are using traditional fuel. In addition to that, this paper aims to study the role of gender and other demographical factors in forming the attitude of users in the Gaza towards solar energy based cooking. Meanwhile, the significance of this study can be summarized as it studies the effect of the gender factor on the use of solar energy for cooking. It also highlights the importance of the gender factor in deciding to use solar energy for cooking in Gaza. Moreover, it presents a comparison between the impact of the financial situation and the gender factor and their role in using solar energy for cooking.

## Impact of awareness and attitude on energy poverty mitigation

According to^14^, Energy poverty, which is the difficulty of having proper access to energy sources including electricity and other energy forms can be mitigated by using renewable energy. The authors of^14^ investigated the impact of using renewable energy on energy poverty. It is claimed by^14^ that the use of renewable energy as well as the careful consideration of renewable energy constrains and prospects would aid in reducing poverty and promoting sustainability in Palestine. This pushed the people of Gaza to think seriously about renewable energy so as to mitigate electricity shortages. It is actually considered as a hope for everyone in Palestine to have their own electricity network. However, the lack of experience in the field of renewable energy at the Palestinian side, the dire need for electricity and the loose energy polices led to a very critical situation.

Currently, the Palestinians are looking forward to find alternative ways of electrification. Thus, the best solution for Israel is to consider this desire seriously^15^. Israel can give access to the 161 kV transmission lines to the Palestinians, while Palestinians can utilize renewable energy sources in generating power. Here, although Israel will lose a huge share of electricity sales, but this can be compensated by wheeling charges. Palestinians will not refuse any wheeling charge as most of the investments will be via donations and loans. After all, Palestinians will not hesitate to pay any charge for electricity independency. On the other hand, the Israeli electricity cooperation will have a much more reliable and stable transmission line with better power flow, power quality and less power losses.

After all, there is a huge renewable energy diffusion that is noticed in Gaza nowadays. In^16^ it is shown that the diffusion of renewable energy installations in Gaza is increased by 730% during the years 2012 and 2019. In this interesting study^16^, the diffusion rate of renewables in the Gaza Strip is investigated against the backdrop of conflict conditions with Israel. According to^16^, Gaza is becoming a renewable energy leader despite conflict conditions exacerbated by deep poverty. The authors of^16^ reported that the balance between discouraging and encouraging factors rests on different variables. These variables are free movement of labor, goods, and fuel, the intensity of the conflict, the role of economies of scale, opportunity costs, and alternative energy production costs. It is also reported in^16^ that most of the recognized systems are solar power systems (either grid connected PV systems or standalone PV systems). Although the authors of^16^ did not discuss the type of renewable energy system, it is clear that the residents of Gaza tend to use familiar renewable energy systems such as standalone PV systems as compared to solar cookers.

In^17^ the authors claimed that renewable energy gaps are attributed to low public awareness. According to^17^, there are two reasons for the gap between the public and renewable energy sources, which are behavioral and structural barriers. Behavior barriers can be simply defined as the negative attitude of the user. Good awareness of renewable energy leads to a positive attitude of users. However, the risk of smart home investments is considered to be one of the behavioral barriers. Furthermore, the lack of the non-technical information about renewable energy systems may cause a negative attitude. On the other hand, it is claimed in^2^ as well that market failure is usually due to the lack of information and low awareness. This study also showed that that the awareness and knowledge of renewable energy is limited.

Previous Research results have also shown that modern energy-efficient electric appliances that are compatible with regional cooking traditions can be greatly desired by customers, in contrast to many Improved CookStoves that have struggled to gain acceptance among consumers^18^. This has the potential to emulate the successes of the mobile phone, mobile money, and solar lighting revolutions that have already swept the Global South, where transformative change was made possible by technological and business model innovations that unlocked dormant consumer demand for aspirational services. However, it is crucial to completely comprehend consumer behavior—not only demands, but maybe more significantly, aspirations—in order to seize these new opportunities. Numerous factors influence consumer behavior, so altering Behavior is intricate^19,20^. As a result, the CookStoves industry has encountered numerous difficulties in its efforts to move consumers toward cooking methods that produce less pollution. Many significant lessons have been discovered that can guide the introduction of eCooking. E-cooking undoubtedly has its own set of problems, but it also provides new approaches to problems that have existed in the past. For instance, it is generally known that a major barrier preventing many people from using CookStoves is the upfront expense of cooking appliances^21^. However, eCooking creates new opportunities for creative financing strategies, such as on-bill financing strategies that let electricity providers sell appliances to their current customers on credit or appliances with locking mechanisms for pay-as-you-go (PAYG) business models that let appliance distributors reach consumers with lower incomes. A key takeaway from the CookStoves implementation programs is that a constant emphasis on technology frequently comes at the expense of the behavior change components required to produce the desired behavioral outcome. In order to better understand these issues and potential, this article will examine consumer behavior in relation to eCooking. It examines how various eCooking solutions fit with the cooking habits of important consumer groups in the Global South, both now and in the future.

All of these researches have stated that renewable energy awareness is a key issue for any technology diffusion. Meanwhile, user’s awareness is associated with many issues such as social and cultural factors including food culture; climatic situation; economic factor and solar cooker price; box cooker quality and durability; accessibility; accessories; operation and maintenance cost and access to information. It is also recommended according to the previously reviewed research papers to test these associations by using a quantitative questionnaire in order to highlight any possible significance between user awareness and the aforementioned factors.

## Research’s questions and hypothesis

Using solar system to obtain electrical energy is an important opportunity in low-income countries, particularly in countries that are located within the solar belt. Thus, moving towards using solar energy to replace traditional energy is an important issue. By reviewing previous studies, it is found that there is lack of research that studies the opinion of Gazan people in using solar energy to provide electricity. Thus, this paper aims to measure the acceptance of people in Gaza strip towards using solar energy systems to power their houses, particularly in operating the cooking appliances.

This research attempts to answer the following main question: “What is the impact of user’s attitude in Gaza Strip on adopting solar cooking appliances?”. Meanwhile, this general question is assumed to be answered by the following questions:

- Does the understanding of using solar energy affect the decision of adopting solar cooking appliances?
- Do the various usages of solar energy in Gaza affect the decision of adopting solar energy cooking appliances?
- Do the attributes of a household in Gaza affect the decision of adopting solar energy cooking appliances?

In this research the significance level is measured at α ≤ 0.05. Based on that, the following hypothesis are assumed in this research,

- There is an association between gender and the use of solar energy in cooking appliances
- There is an association between the level of education and the use of solar energy in cooking appliances
- There is an association between age and the use of solar energy in cooking appliances
- There is an association between the number of people living in the same house and the use of solar energy in cooking appliances
- There is an association between employment type and the use of solar energy in cooking appliances
- There is an association between place of residence and the use of solar energy in cooking appliances

## Research’s methodology and statistical analysis plan

This research follows descriptive and analytical approaches in order to answer the aforementioned research question. The study population consists of the employees of three local universities in Gaza which are about 2400 employees. This study includes respondents who installed solar energy systems at their houses (12%). An electronic questionnaire is used in this research, where, 342 respondents out of 2400 have responded to the questionnaire. This response actually represents the sample population with a margin of error of 5% and confidence level of 95%.

The researchers recorded and analyzed the questionnaire data by using the statistical analysis program "Statistical Package for the Social Sciences (SPSS)". The following statistical methods have been used.

- Descriptive statistics, including: Percentage, Mean, Standard Deviation, and Relative Weight. These factors are used mainly in order to measure the repeated class of a variable and to help the researchers to describe the study variables.
- Pearson Correlation Coefficient: To verify the reliability of the internal consistency between the items of the questionnaire and the total score of the questionnaire.
- Cronbach's Alpha Coefficient: To find out the stability of the questionnaire sections.
- Spearman's Correlation Coefficient for equal half segmentation: To find out the stability of the sections of the questionnaire.
- Normality distribution test: Kolmogorov–Smirnov test, to find out whether the data are subject to the normal distribution or not.
- Independent sample T-Test: To find out if there are significant differences between two sets of ordinal data.
- One- Way ANOVA Test: To find out if there are significant differences between three or more sets of ordinal data.
- LSD Test: To know the nature of the differences.

### Ethical statement

All experimental protocols were approved by the ethical research committee at the Islamic University of Gaza and all methods were carried out in accordance with relevant guidelines and regulations. The committee members are Prof. Dr. Adel M. Awadallah and Prof. Dr. Yousef I. Al-Jeesh. Also, informed consent was obtained from all subjects included in the study.

## Results and discussion

### Description of the study sample

This subsection presents the distribution of the sample individuals according to different attributes (gender, academic level, age, number of family members in the house, employment type, and the type of residence). In the selected sample, 56% are males while 44% are females. This percentage represents somehow the gender demography is at Gaza’s universities.

As for education, the participants in this study do have postgraduate degree (58%), while most of the remaining participants do have Bachelor’s degree (31%). On the other hand, the analysis of participants’ age showed that 47% of the sample are above 40 years. Meanwhile, 28% of the respondents’ age is in the range of (30–35) years, while 25% of them are below 30 years. As for family size, most of the participants have a large family size, where 87% participants have more than 4 family members, while 15% of them have 3 to 4 family members. The occupation type is also considered when analyzing the sample, where more than half the sample (54%) hold administrative positions, while, 35% of them hold academic positions. In addition to that, 72% of the respondents are living in owned residences, while 18% are still living in their parents’ houses (shared residences), and 10% of the participants are living in rented residences. Finally, it was found that 86% of the respondents have enough space to install solar panels. Moreover, it was also found that 54.6% of the participants already have solar systems in their houses, whereas 47.7% of these systems were installed between 2014 and 2018, 40.9% of these systems were installed 2018, and 11.4% of these systems were installed before 2014.

### Statistical analysis results

In this part, the respondents are categorized according to their knowledge in solar energy usages. Table 1 gives a summary regarding the results of eight different items that are meant to measure participants’ knowledge of solar energy system usages using arithmetic mean (A.M.), standard deviation (S.D.) and the relative weight% (R.W.%).

**Table 1:** Participants knowledge about solar energy assessment by using A.M., S.D. and R.W measures.

No.	Item	A.M	S.D	R.W. %	Rank
1	You have initial knowledge about the uses of solar energy	8.023	2.445	80.23	2
2	Solar energy is used for lighting	9.455	1.438	94.55	1
3	Solar energy is used for water desalination	5.773	4.063	57.73	4
4	Solar energy is used for wastewater treatment	4.159	3.748	41.59	7
5	Solar energy is used for operating electrical devices	7.523	2.905	75.23	3
6	Solar energy is used for cooking	3.773	3.333	37.73	8
7	Solar energy is used to operate large facilities such as (factories, universities, hospitals)	5.273	3.979	52.73	5
8	Solar energy is used for heating and cooling devices	4.273	3.493	42.73	6
	Overall Average	6.031	3.175	60.31	

The results tabulated in Table 1 show a good knowledge of solar systems among users. Among different usages of solar systems, respondents believe that the best usage of solar energy is for lighting with a relative weight of 94.55% followed by electrical devices with a relative weight of 75.23%. Meanwhile, according to the results of Table 1, the lowest three ranked items are heating and cooling devices with a relative weight of 42.73%, then wastewater treatment with relative weight of 41.59% and finally cooking has the least relative weight of 37.73%. This supports the importance of this study to disseminate the importance of solar energy systems for powering cooking devices.

Further investigation of respondents opinions on the most suitable method for using solar power cooking appliances shows that only 19.7% believed that direct solar energy is a suitable method for cooking, while 54.1% of the respondents voted for solar photovoltaic systems and 26.2% of the respondents voted for sun baths.

In addition to that, investigation is done to describe the participant’s response according to their choices to use solar system in their homes particularly in the kitchen as displayed in Table 2.

**Table 2:** Distribution of participant’s response to the usage of solar energy in the kitchen.

No.	Item: solar energy is used in kitchen for	A.M	S.D	R.W. %	Rank
1	Cooking	4.614	3.200	46.14	4
2	Preparation of hot drinks	4.023	3.514	40.23	6
3	Cleaning	5.159	3.464	51.59	3
4	Powering electric devices (light bulb, radio, and air conditioning)	7.023	2.749	70.23	2
5	Powering refrigerator	8.000	2.779	80.00	1
6	Powering microwave	3.795	3.548	37.95	7
7	Powering dish washer	4.045	3.660	40.45	5
	Overall Average	5.237	3.273	52.37	

From Table 2, the highest ranked use of solar energy in kitchen was for powering refrigerators, with a relative weight of 80%, followed by powering electric devices with a relative weight of 70.23%, while cleaning comes third with a relative weight of 51.59%. The reason behind that is frequent power shortage in Gaza. During power shortage people seriously care about food inside refrigerators and usually use small generators to power these fridges in order not to lose the food inside it. Thus, there is a of alternative power source to power fridges in Gaza and that is why respondents strongly believe that solar energy can be used for refrigeration. In Table 2, the least ranked usages of solar energy is for powering dish washers with a relative weight of 40.45%, followed by water kettle with a relative weight of 40.23%, while using solar energy to power microwaves got the least attention with a relative weight of 37.95%.

In order to study the impact of the financial situation on respondents’ decision to adopting solar energy systems at their homes, particularly for powering kitchen appliances, the A.M., S.D., and the R.W measures were also used. Table 3 displays the impact of the financial situation on adopting solar systems to power electrical equipment at households. According to Table 3, the highest ranked item is item number (9) which the option of paying the capital cost of solar energy system as instalments without profit, with R.W. of 86.95%. This indicates that the financial situation has an important effect on the decision to adopt solar energy. Next highest item is item number (7) which is about the profits and savings that can be earned by solar energy systems, with R.W. 82.73% which assures that the study sample individuals adopt solar systems because they believe that it is feasible. The following highest item is item number (8) which is about of high electricity bills that motivate the people to use alternative energy with R.W. 82.27% which again supports the idea that the readiness of people to adopt solar energy is affected by the financial situation. However, item number (2) “The increase in the price of solar energy over traditional energy methods plays a role in not adopting solar energy.” with R.W. 60.91% scored the least. This may indicate that the study sample individuals do not think that the price of the system affect their decision as long as they still can save money after installing the system. Moreover, the energy critical situation makes the users more open for medium investment in order to enhance the quality of service. This point is supported by item number (6) “In case the cost of solar energy and conventional energy are equal, will you choose solar energy?” with R.W. 77.05%.

**Table 3:** Impact of the finical situation on making decision to use solar energy at home.

No.	Item	A.M	S.D	R.W. %	Rank
1	The effect of your income level on your choice of the power source you are using	7.750	2.721	77.50	7
2	The increase in the price of solar energy over traditional energy methods plays a role in not adopting solar energy	6.091	3.109	60.91	9
3	Governmental support (if found) for solar energy prices pushes you toward adopting it	8.205	2.882	82.05	4
4	The cost of solar energy is lower than that of traditional energy at the long term	7.909	2.640	79.09	6
5	The lack of traditional energy due to the political situation pushes you toward adopting the solar energy	7.932	2.823	79.32	5
6	In case the cost of solar energy and conventional energy are equal, will you choose solar energy ?	7.705	3.100	77.05	8
7	You think that using solar energy saves money	8.273	2.106	82.73	2
8	Do you think that the monthly electricity bills affect you financially, so you want to use renewable energy as an alternative?	8.227	2.166	82.27	3
9	Do you think that companies that supply solar energy systems should accept payment in installments	8.659	2.145	86.59	1
	Overall Average	7.861	2.632	78.611	

Table 4 displays the items that affect the decision of the study sample individuals to install solar systems. It shows that the most three items that affect the decision are; item number (9) “If you got a convenient funding, does it help you to decide to install a solar system?” with R.W. 90.23% which second our previous findings that the financial status plays a role in adopting solar systems. This choice is followed by item number (5) “Do you prefer to use solar energy as a green renewable energy resource?” with R.W. 88.64% and item number (10) “Do you agree that the society in Gaza needs more knowledge and awareness of renewable energy?” with R.W. 87.73%. This shows that the level of awareness of solar energy technology is not that high in Gaza and awareness campaigns are still required.

**Table 4:** Main reasons that affect decision to adopt solar systems.

No.	Item	A.M	S.D	R.W.%	Rank
1	The scheduled cut-off of electricity due to increased loads has a negative effect on you at home	8.227	2.477	82.27	6
2	Renewable energy technologies are not widely distributed in Gaza’s local market	5.455	2.698	54.55	10
3	The renewable energy sector is still weak in Gaza Strip	6.227	2.514	62.27	8
4	Do you think that future of solar energy is to replace traditional energy?	8.136	2.007	81.36	7
5	Do You prefer to use solar energy as a green renewable energy resource?	8.864	1.760	88.64	2
6	The easiness of installing solar cells and the availability of spare parts and maintenance contributes in adopting it	8.614	1.728	86.14	5
7	Living in a rural place pushes you toward adopting solar energy	5.909	3.354	59.09	9
8	The availability of solar energy throughout the year pushes you toward adopting solar energy	8.705	1.786	87.05	4
9	If you got a convenient funding, does it help you to decide to install a solar system?	9.023	1.785	90.23	1
10	Do you agree that society in the Gaza needs more knowledge and awareness of renewable energy?	8.773	1.597	87.73	3
	Overall Average	7.793	2.170	77.93	

The least items that affect the study sample’s opinion to adopt solar systems is item number (2) “Renewable energy technologies are not widely distributed in Gaza’s local market.” With R.W. 54.55% which means that the lack of technology in the market does not affect their decisions.

After studying the sample statistical distribution and reasons that might affect the sample decision in adopting solar system, the assumption of the current study are tested as below.

It in this research it is assumed that there are statistically significant differences at the significance level (α ≤ 0.5) between average degrees of using solar energy in cooking tools among respondents attributed to gender. To verify the assumptions "Independent sample T-test" is used to measure the difference between the two variables.

In this research four factors are used to judge the perception of using solar energy cooking systems, which are “knowledge of using solar energy”, “the use of solar energy in the kitchen”, “financial situation”, and “decision making” later called (The average degree of using solar energy for cooking).

Table 5 shows the value of significance level considering gender. According to the table, there are no statistically significant differences at significance level 0.05 between average degrees of using solar energy for cooking.

**Table 5:** Results significance level for Gender.

	Gender	Arithmetic mean	Standard deviation	"T" test value	Significance level
Knowledge of using solar energy	Male	6.136	2.310	0.579	0.566
	Female	5.675	1.824		
The use solar energy in the kitchen	Male	5.172	2.560	0.301	0.765
	Female	5.457	2.862		
Financial situation	Male	8.023	1.273	1.025	0.327
	Female	7.311	2.085		
Decision making	Male	7.876	0.997	0.633	0.450
	Female	7.510	1.749		

On the other hand, Table 6 shows that there are statistically significant differences at significance level between the average degree of using solar energy for cooking attributed to the educating level. This is quite expected as education and knowledge are directly associated and this consequently affect the decision making.

**Table 6:** Results significance level for educational level.

	Postgraduate	Bachelor degree	Diploma	High school	Others	F test value	Significance level
Knowledge of using solar energy	5.534	6.025	8.031	8.937	3.250	2.779	0.040
Using solar energy in the kitchen	5.019	5.028	5.464	8.000	6.417	0.696	0.600
The use solar energy in the kitchen	7.788	7.459	8.694	9.222	9.444	1.323	0.279
Decision making	7.800	7.327	8.725	9.300	7.900	2.198	0.078

Table 7 also shows some significant differences between average degrees of using solar energy in cooking attributed to their age especially knowledge of solar energy and financial situation. The situation is also expected as age reflects knowledge and financial potential especially in young respondent’s cases. On the other hand, Table 8 shows that there are no statistically significant differences between average degrees of using solar energy in cooking tools attributed to their job status.

**Table 7:** Results significance level for age.

	Less than 35	35–45	More than 45	F test value	Significance level
Knowledge of using solar energy	5.589	7.104	5.640	2.066	0.140
Using solar energy in the kitchen	5.489	5.785	4.903	0.495	0.613
The use solar energy in the kitchen	7.159	8.759	7.627	3.626	0.035
Decision making	7.243	8.200	7.752	1.486	0.238

**Table 8:** Results significance level for career type.

	Administrator	Academic	Others	F test value	Significance level
Knowledge of using solar energy	6.083	5.687	7.021	0.852	0.434
Using solar energy in the kitchen	4.960	5.271	5.952	0.320	0.728
The use solar energy in the kitchen	3.704	7.911	8.167	0.227	0.798
Decision making	7.694	7.835	7.950	0.120	0.887

## Conclusion

In this paper the perception of using solar energy cooking appliances in Gaza Strip was investigated. The study sample population was 2400 employees in the universities of Gaza. 347 participants have participated in the study which gave the results a margin of error of 5% and a confidence level of 95%. Different attributes were used to measure the perception of the respondents to solar energy cooking systems including knowledge of using solar cooking appliances, financial situation, educational level, age, career, type of living place and gender. Results showed that participants believe that the best usage of solar energy is for lighting with a relative weight of 94.55% while cooking has the least relative weight of 37.73%. It is also concluded that there are no statistically significant differences at significance level (smaller than 0.05) between average degrees of using solar energy for cooking (knowledge of using solar energy, using solar energy in the kitchen, financial situation, and making decision of using solar energy in the kitchen) attributed to gender and job status. Meanwhile, it was found that there are statistically significant differences between the average degrees of using solar energy for cooking attributed to participants’ education, and age. The result is understandable since education and age highly affects our decisions.

In addition to that the authors have concluded some remarks regarding the use of solar energy based on this study as follows,

- For end-users who have middle income, the affordable price of technology is always important for using this technology, but is not in comparison with quality of life
- The role of “seeing is believing” is not yet achieved with regards e-cooking in Gaza as most of the respondents act toward this technology considering solar PV which is a different technology. Thus, it is important to fund some pilot project first before doing market assessment.
- Peer to peer marketing is very important in Gaza considering the common needs
- Funding schemes and loans are key issues in spreading the e-cooking technology
- When it comes to daily need, education level does not play that much role in choosing novel technologies such as e-cooking
- The people of Gaza do not differentiate between different uses of solar energy in Gaza regardless educational level
- The large support of using solar energy in general is directly associated with energy poverty status in Gaza
- In general, the idea of using green alternative energy is acceptable in Gaza but there is a clear lack of awareness of technologies aspects and characteristics

## Data Availability

The datasets used and/or analysed during the current study available from the corresponding author on reasonable request.

## Abbreviations

E-cooking: Electrical based cooking
SPSS: Statistical package for the social sciences
A.M: Arithmetic mean
S.D: Standard deviation
PV: Photovoltaic
α: Significance level
PAYG: Pay-as-you-go
R.W.%: Relative weight
